# Fresh Pod Yield, Physical and Nutritional Quality Attributes of Common Bean as Influenced by Conventional or Organic Farming Practices

**DOI:** 10.3390/plants12010032

**Published:** 2022-12-21

**Authors:** Ioannis Karavidas, Georgia Ntatsi, Sofia Marka, Theodora Ntanasi, Beppe Benedetto Consentino, Leo Sabatino, Pietro P. M. Iannetta, Dimitrios Savvas

**Affiliations:** 1 Laboratory of Vegetable Crops, Department of Crop Science, School of Plant Sciences, Agricultural University of Athens, 75 Iera Odos, 11855 Athens, Greece; 2 Laboratory of Cell Technology, Department of Biotechnology, School of Applied Biology and Biotechnology, Agricultural University of Athens, 75 Iera Odos, 11855 Athens, Greece; 3 Dipartimento Scienze Agrarie, Alimentari e Forestali (SAAF), University of Palermo, Viale delle Scienze, Ed. 5, 90128 Palermo, Italy; 4 Ecological Sciences, James Hutton Institute, Invergowrie, Dundee DD2 5DA, UK

**Keywords:** *Phaseolus vulgaris* sp., organic fertilization, antioxidant activity, phenolics, flavonoids, carbohydrates, nitrogen

## Abstract

The aim of the current study wat to comparatively assess the impact of different nitrogen (N) fertilization schemes on fresh pod yield and yield quality in either organically or conventionally grown common beans (*Phaseolus vulgaris* L.). Prior to common bean crop establishment, the experimental field site was cultivated following either organic (a) or conventional (b) farming practices with a winter non-legume crop (*Brassica oleracea* var. *italica*) (BR), or (c) with field bean (*Vicia faba* sp.) destined to serve as a green manure (GM) crop. At the end of the winter cultivation period the broccoli crop residues (BR) and green manure biomass (GM) were incorporated into the soil and the plots that accommodated the treatments (a) and (c) were followed by an organically cultivated common bean crop, while the conventional broccoli crop was followed by a conventionally cropped common bean crop. Additional to the plant residues (BR), sheep manure (SM) at a rate of 40 kg N ha^−1^ was also applied to the organically treated common beans, while the plots with a conventionally cropped common bean received 75 kg N ha^−1^. Organic common bean treated with SM + BR produced smaller pods of higher dry matter and bioactive compound content, responses that are correlated with limited soil N availability. No significant variations were observed on yield components and N levels of pods cultivated under organic (SM + GM) and conventional cropping systems. Pod sugar and starch content was not influenced by the different fertilization practices. In conclusion, we have demonstrated that the combined application of SM + GM can be considered as an efficient N-fertilisation strategy for organic crops of common bean, benefiting their nutritional value without compromising yield.

## 1. Introduction

Extensive application of inorganic nitrogen (N) is considered one of the most important factors contributing to elevated GHG emissions and pollution of surface- and groundwater with nitrates [1,2,3]. Historically, high inputs of inorganic fertilizers and agrochemicals have been considered as acceptable approaches to ensure and safeguard crop production in conventional agriculture [4]. However, the application of synthetic agricultural chemicals for pest and weed control can be detrimental to both the environment by degrading the biodiversity in agriculture areas [5,6] and human health through either occupational exposure [7,8] or consumption of contaminated products [9]. Seeking solutions with the aim to achieve healthier products through eco-friendly production systems, organic cultivation practices can be considered as a solution since inorganic N and synthetic agrochemicals are avoided. According to Gatsios et al. [10,11,12,13], N supply in organic agriculture mainly relies on green and animal manures, composts, crop rotation with legumes, and utilisation of intercropping approaches. The introduction of the above-mentioned renewable N sources into agricultural production systems can also reduce contamination of water resources with nitrates [14] and increase the diversity of soil microbiota [15], thereby fostering soil nutrient availability, productivity, and health [16].

Despite the application of policies to increase the uptake of organic farming, organic growers represent only a small proportion of the farming community. The reluctance of farmers to turn to organic agriculture is mainly attributed to the 20–30% lower productivity of this system compared to the yields obtained from conventional farming systems. Furthermore, the reduced productivity in organic crops is seen as a food- and feed-security risk, that would also manifest in higher prices for purchasers [16,17]. According to Seufert et al. [17] this yield gap is mainly ascribed to inappropriate timing in N supply, rather than in reduced total input of N.

Nowadays, organically produced crops are receiving ever-increasing attention from consumers around the world, especially in more affluent countries. This may be ascribed to the fact that many consumers perceive organic farming as a more environmentally responsible production process offering produce of higher nutritional and organoleptic value. Particularly, according to many reports, the organic-labelled plant products exhibit higher nutrient content with restricted nitrate accumulation [18,19], and higher amounts of fiber and antioxidant compounds [20,21]—in addition to avoided exposure to man-made pesticide residues.

Common bean (*Phaseolus vulgaris* sp.) is a vegetable rich in nutrients, proteins, amino acids and carbohydrates [22,23], while its hulls exhibit an advanced antioxidant and anticancer activity [24]. The common bean is also the most popularly human-consumed grain legume globally [25]. Being a legume, the common bean forms a symbiotic association with the soil microbes collectively termed ‘rhizobia’, to acquire physiologically useful N forms from atmospheric N_2_ via ‘biological nitrogen fixation’ (BNF). However, due to its poor N_2_ fixing activity [26], common bean productivity relies mainly on external N inputs [27] in contrast to most other legume crops. Karavidas et al. [28] highlighted several N fertilization practices that decrease the dependence of the common bean on external N input while safeguarding yield, especially in organic farming systems where soil N availability is the main limiting factor for crop productivity. Unlike the yield responses of common bean to N fertilization, there is a lack of knowledge concerning the effects of such organic N inputs on common beans cultivated for green pod production, and the nutritional qualities of the pods produced.

The objective of this study was therefore to investigate the impact of organic fertilization strategies of different preceding crops (during the winter) on the yield and yield attributes of common bean green pods in the subsequent organically cultivated common bean crop. The crop rotation of a winter non-legume crop with a summer common bean crop was also tested under conventional fertilization management. During the winter cultivation period, broccoli (*Brassica oleracea* var. *italic*), a vegetable with high N requirements [29,30,31], was cultivated as a non-legume crop, while faba bean (*Vicia faba* sp.) was chosen for a green manure crop due to its advanced BNF ability, and high N contribution [32]. As the beneficial effects of the crop rotation appear over the long term [33], the above rotation schemes were repeated during the following year and the quality assessment was conducted on the common bean fresh pods obtained only from the second experimental year.

## 2. Results

### 2.1. Yield Components and Morphological Characteristics of Green Pods

The quality of the fresh common bean pods was affected by the different farming practices as statistically significant differences were observed in almost all quality characteristics assessed (Table 1). Cultivation of faba beans (SM + GM) increased the number of pods per plant (NPP), the mean fresh pod weight (MFW), as well as the length (PL), suture string (SS), width (PW), and seed number per pod (SNP) by 16.3, 3.2, 9.8, 5.2, 3.6, and 10.8%, respectively, in the subsequent organic crop of common beans, compared to the cultivation of broccoli as a preceding winter crop. Also, under organic farming practices (SM + BR), the dry matter content (DMC) of the green pods increased by 12% when the preceding winter crop was broccoli compared to the cultivation of faba bean as a green manure during the winter. The conventional cultivation of common beans resulted in pod characteristics (NPP, MFW, PL, SS, PW, SNP, DMC) which were not significantly different from that recorded for the organic cropping system, when faba beans were cultivated as GM. The yield components and the morphological quality characteristics of common bean pods were not significantly influenced by any of the treatments tested in the current study.

### 2.2. Nutrient Content of Fresh Pods

**Figure 1:**
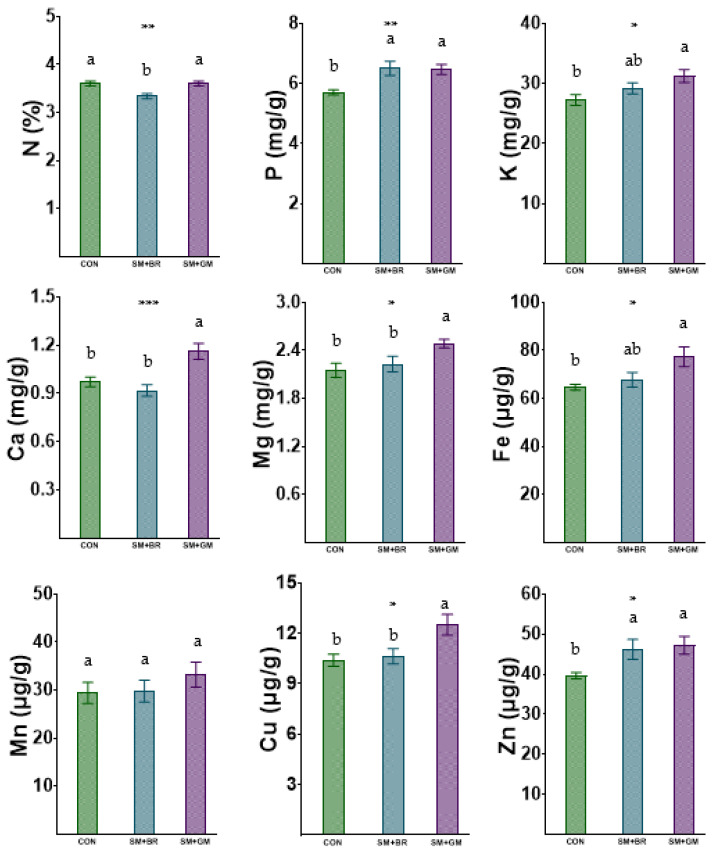
Impact of the three different farming practices on macro-(mg/g) and micronutrient (μg/g) concentrations in common bean pods (on DW basis). Different letters above each bar within the same graph indicate statistically significant differences according to the Duncan multiple range test at *p* < 0.05. *, ** and *** denote significant differences at *p* < 0.05, 0.01 and 0.001 respectively. CON denotes conventional farming, SΜ denotes sheep manure, GM denotes green manure and BR denotes broccoli residues.

Organic cultivation of common beans based on the cultivation of faba beans (SM + GM) during the preceding winter resulted in similar total N concentrations in green pods with those recorded in conventionally produced pods, but the total N level in green pods produced organically after a winter crop of broccoli (Figure 1) was significantly lower. Furthermore, green manure (SM + GM) application increased the Ca, Mg and Cu concentrations in organically produced pods of common beans compared with both organic common bean cultivation following an organic broccoli crop (SM + BR), and conventional cropping. On the other hand, organic cultivation of common beans increased the P and Zn concentrations in green pods compared with conventional production, irrespective of GM application or not. The P and Fe concentrations were also higher in green pods produced organically compared to conventional cropping, but the differences were significant only when the organic fertilization included faba beans (SM + GM). Finally, the pod Mn concentration was not influenced by the farming system, or the application of GM in the organic farming treatments.

### 2.3. Biochemical Compounds

In the current study, the antioxidant activity of dry common bean pods was displayed by two different methods, namely, the ferric reducing antioxidant power and the Trolox equivalent antioxidant capacity. Both methods revealed that the antioxidant potential of pods produced organically without green manure application (SM + BR) was significantly higher than in pods produced in the conventional (CON) cultivation system (Table 2). However, organic cropping with green manure application (SM + GM) had no significant impact on the total antioxidant activity in green pods compared to conventional cropping. The application of GM in organic cropping had no impact on the total antioxidant activity but reduced the total phenolic and flavonoid content significantly compared to organic farming of common beans after an organic broccoli crop, but not after a preceding winter GM application.

### 2.4. Sugars and Starch

The conventional and the two different organic fertigation management practices did not influence the carbohydrate content of fresh common bean pods (Table 3). In particular, the applied enzymatic assay did not reveal any significant variation between the different treatments to both sugars and starch content.

### 2.5. Principal Component Analysis (PCA)

**Figure 2:**
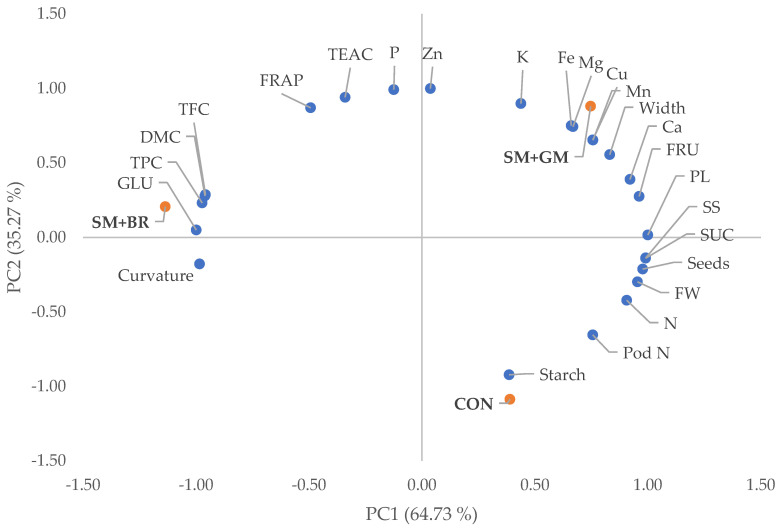
Scores and loading plots of common bean physical traits (number per plant (Pod N), fresh weight (FW), length (PL), suture string (SS), curvature, width and seed number of pods) and nutrient (N, P, K, Ca, Mg, Fe, Mn, Cu and Zn), bioactive compound (FRAP, TEAC, total phenolic (TPC) and total flavonoid (TFC) content), sugar (glucose (GLU), fructose (FRU) and sucrose (SUC)), and starch content of pods. CON denotes conventional farming, SΜ denotes sheep manure, GM denotes green manure and BR denotes broccoli residues.

The PCA of physical and chemical quality parameters of fresh common bean pods derived from different cropping systems indicated that the first two principal components (PCs) clarified 100% of the total variance with PC1 and PC2, resulting in a percentage of 64.7 and 35.3%, respectively (Table S1). PC1 was positively correlated with the nutrient profile (except for P content), pod yield components (except for curvature and %DMC) and fructose, sucrose and starch content of common beans pods, while PC2 was negatively correlated with most yield components (except for pod width, length and %DMC) and total N), sucrose and starch content of pods. According to the graphical representation of PC1/2 (Figure 2), organic beans treated with both animal and green manure (SM + GM) are placed on the upper right quadrat, while the left upper quadrat outlines organic common beans treated only with SM and broccoli crop residues (SM + BR). Finally, the lower right quadrat contains common beans cultivated under standard inorganic fertigation management (CON).

## 3. Discussion

As N availability is the main factor limiting productivity in organic vegetable cropping systems, Schmeer et al. [34] claimed that legume crops, due to their BNF activity, can overcome the above limitation and perform as efficiently as crops treated with inorganic N fertigation schemes. However, this was not the case in the present study, where the organic common bean crop (SM + BR) restricted the number and size of fresh pods, and hence total fresh pod yield. This is mainly ascribed to the fact that the productivity of common bean crops relies on external N input, due to its poor capacity to fix atmospheric N_2_ [27]. The restricted N availability for common beans cultivated organically without GM (SM + BR) is indicated by reduced total N concentrations in pods produced. Similar yield responses of common beans to sub-optimal soil N availability have also been reported by Chekanai et al. [35], Elkhabit [36], Karasu et al. [37], and Silva et al. [38], who cultivated crops for either green pods, or dry seeds. The beneficial effect of GM application for organically grown common yield seen here, is in accordance with the study of Gatsios et al. [12], who also found that the incorporation of animal manure and GM, including faba beans, enhanced the yield of a subsequent tomato crop. Contrary to the above findings, in the study of Kontopoulou et al. [3] GM application did not mitigate the fresh pod yield gap between organic and conventional common beans. This is mainly ascribed to the fact that in the study of Kontopoulou et al. [3] both the organic and the conventional common bean crops were treated with GM.

According to Lairon [21], organic farming practices are of more benefit to the mineral profile of vegetables compared to fruit and grain crops. These benefits can be ascribed to both soil nutrient availability and plant nutrient uptake. Particularly, the elevated soil availability of organic compounds, as a function of the higher microbial activity of organically amended soils, can benefit plant P, Fe and Mg uptake [39]. Additionally, soil acidification which results from the higher levels of soil organic matter, can enhance nutrient solubility [40]. However, in the present study the quality, in terms of mineral profile, of organic common bean pods (SM + BR) did not substantially differ from that obtained under conventional farming practices, as the organic pods recorded an increase in P and Zn concentrations only compared to the conventionally produced pods. The trend of recording restricted N and enhanced P concentrations in organic vegetable products has already been reported in the study of Dangour et al. [41]. Conversely, the organic cultivation system involving GM application produced pods with higher mineral content compared to those obtained under inorganic and organic cropping without GM application. Thus, the beneficial effects of organic fertilization managements reported here were seen in crops treated with both SM and GM inputs. This confirms the notion that the yield limitation often associated with organic crops is largely due to inadequate N availability at critical growth stages and can be overcome by applications which ensure N needs are met throughout the cropping period. In organic crops of common beans under Mediterranean climatic conditions, a combination of animal manure application with green manuring by incorporating a winter legume crop to the soil can adequately cover the crop N needs thus providing similar yields with conventional crops. More specifically, animal manure can meet crop needs at the earlier cropping stages, as it is quickly mineralized in the soil [42], while the GM provides N to the crop at later cropping stages due to a slower and more uniform mineralization rate [43], when the contribution of animal manure to plant N supply has diminished to sub-optimal levels.

In the current study, the organically grown winter broccoli diminished soil fertility for the following common beans. In contrast, the faba bean crop did not deprive the subsequent common bean crop of any nutrients, as it was incorporated to the soil as GM, while it provided additional N to the crop originating from symbiotic N_2_. Nevertheless, the yield and quality of conventionally cultivated common beans were similar to common beans treated with both SM and GM inputs. It therefore appears that the mineral fertiliser N application to conventionally cropped common beans compensated the poor soil fertility following winter broccoli. These results suggest that the best practice in organic cultivations of common bean under mild winter conditions is to avoid a second vegetable crop during the winter, and instead grow a legume such as faba bean as a GM. Growing successively a winter and a summer vegetable during the same year is too intensive a rotation scheme for organic cropping systems, which would have the reserves of available soil N depleted, and the application of SM would not be sufficient to replace all N needed for the two crops.

The increased P concentrations in organically produced green beans may be due to greater P soil availability, as the application levels of SM used to meet the crop N requirements provides surplus P [44]. However, this may also be due to a more efficient transport of P to the root system as a function of higher microbial activity and specifically of beneficial microorganisms, such as mycorrhiza [45].

In the present study, the common bean plants were cultivated for fresh pod production. However, the pod nutrient content was standardized on dry weight basis. Thus, the higher DMC of fresh pods cultivated organically demonstrates a higher nutrient content compared to the conventionally grown pods, even though the nutrient concentrations expressed on dry weight basis are the same. Dry matter content is considered a quality trait, closely related with the organoleptic value [46] and post-harvest shelf-life of plant products [39], and a trait which is improved by organic fertilization regimes [21,47]. According to Herencia et al. [48], their differential chemical composition, as inadequate N availability in organically treated soils promotes the synthesis of N-poor alternative molecules. In agreement with this observation and theory, Bourn and Prescott [49] concluded that the excessive N fertilization in conventional cultivation systems results in greater moisture content in the vegetables produced. In the present study, the DMC of common bean pods also correlated with N supply as opposed to specific agronomy (i.e., organic or conventional). This agrees with the notion that the N availability for the plants is the ultimate factor that determines the DMC in plant tissues. Indeed, enhanced DMC was only recorded in organic beans fertilized with only SM, which resulted in N malnourishment. An inverse relationship between N availability and DMC in fresh pods has also been reported in an experiment with common beans cultivated either in an organic [3] or in a soilless [50] cultivation systems.

Antioxidants, phenolics, and flavonoids represent three major groups of secondary metabolites in plants that are highly benefited by organic cultivation managements [16,20,21,51,52,53]. According to the studies of Faller and Fialho [54] and Magkos et al. [55], the restricted availability of N and other nutrients in organically treated soils limits the growth rate of the plants, resulting in the synthesis of antioxidants and phenolic compounds. Moreover, stresses induced by the inability of organic farming practices to cope with abiotic factors, such us weed and pest pressure, also promotes the production of these bioactives [54,56]. However, the plant protection practices in the current study were similar in both the conventional and the organic treatments, to discern only the impacts of the different N fertilization practices. This was facilitated by the lack of disease and pest problems in the experiments. Given this, the above-referenced reports are in full agreement with the current study, as higher levels of such bioactive compounds were recorded in pods obtained from (SM + BR)-treated plants, where restricted N content was also observed. Similarly to the current study, Mastura et al. [57] also reported that organic cultivation favored the antioxidant and phenolic content of Borlotti beans compared to conventional cropping.

As stated by Lairon [21], there is limited data concerning the impact of organic cultivation on the sugar and starch content in plant products. According to Rembiałkowska [39], organic plant products are expected to contain more starch, a low-N-containing molecule, as a response to the limitations in soil N availability. Furthermore, Conti et al. [58] reported an increase in glucose and fructose contents in organic strawberries compared to conventional ones, while Maggio et al. [59] observed an inverse relation between the N fertilization regime and the sugars and starch content in potato tubers. Several researchers suggest that the sugar content of organic vegetables and fruits depends on both the genotype and the environmental conditions [60,61,62]. However, in the current study the different N fertilization treatments had no impact on the sugar and/or starch content of common bean pods. This finding might be ascribed to the considerably lower N requirements of common bean plants in comparison with the non-BNF species evaluated in the studies referenced above. Consequently, we can presume that either the restricted soil N availability in SM-treated plants was not severe enough to affect the sugar content of the produced pods, or the bean genotype cultivated in the current study did not respond differently, in terms of carbohydrates content, under organic and inorganic fertilization managements. To the best of our knowledge, there are no published studies reporting the impact of different N input regimes on the carbohydrate profile of common bean green pods.

## 4. Materials and Methods

The field experiment was conducted during autumn 2017 to summer 2018 and repeated the following year (autumn 2018 to summer 2019) at the experimental facilities of the Laboratory of Vegetable Production at the Agricultural University of Athens. Given that the beneficial effects of rotation schemes appear in the long term, the impact of the different cultivation systems on quality of the common bean pods was evaluated only in the second experimental year (autumn 2018 to summer 2019). In both years the experimental organic field with a total area of 375 m^2^ was divided into 24 farming plots of 5 m^2^. The soil type of the field was sandy loam, and its physicochemical properties are presented in Table 4. In addition, climatic data for the experimental period, particularly monthly temperatures (minimum, average minimum, maximum, and average maximum) and monthly mean precipitation, are presented in Table S2.

### 4.1. Experimental Design

The 24 experimental plots were divided into 8 blocks with three plots per block, representing three different fertilization treatments. During the winter cultivation period the cropping and fertilization practices applied in each of the three plots per block were: (i) broccoli for commercial trunk production following organic farming practices, (ii) broccoli for commercial trunk production following conventional farming practices and (iii) faba bean destined for application of green manure. During the subsequent summer cultivation period, the plots that accommodated faba bean for green manure and organic broccoli during the winter were followed by organic farming of common bean, while the plots used to grow broccoli conventionally during the preceding winter were followed by conventional farming of common bean. An overview of the treatments during both cultivation periods is provided in Table 5.

### 4.2. Cultivation Practices

*Vicia faba* sp. was chosen for the establishment of green manure crop as a cold season legume characterized by high nitrogen fixing efficiency. The selected variety of faba bean originated from the Greek island Lefkada. The green manure crop was established on 30 October 2018 by sowing faba bean seeds at a plant density of 30 seeds m^−2^. Furthermore, as a low input fertigation scheme is consider to promote plant growth at the early growth stages, where the nitrogen fixing activity of legumes is not developed yet, sheep manure (0.84% total N, 0.3% P_2_O_5_, 0.7% K_2_O, 0.38% CaO, and 0.24% MgO, on dry weight (DW) basis), which provided 40 kg ha^−1^ N, and patent kali (30% K_2_O, 10% MgO and 42.5% SO_3_, K + S AG) at a rate of 400 kg ha^−1^ were applied as basal dressing. At the end of February 2019 when the faba bean plants reached their 50% flowering stage, the crop was incorporated into the soil. The conventional and organic broccoli crops were planted on 30 October 2018. For broccoli cultivation the one-head variety named Monrello was selected. In the conventional broccoli, the whole inorganic fertigation scheme provided 239.2 kg N ha^−1^, while in the organic broccoli the application rate of sheep manure as basal dressing provided 120 kg N ha^−1^ during the winter cultivation period, respectively. On February 2019, the broccoli crops, irrespective of the farming system, were terminated and then the broccoli plant residues (BR) were incorporated into the soil. To facilitate incorporation, both the faba bean plants (GM) and broccoli crop residues (BR) were cut with a flail mower, and then the chopped plant biomass was incorporated into the soil by plowing.

During the successive summer cultivation period the experimental field was cultivated with a climbing variety of common bean (*Phaseolus vulgaris* cv. Borlotto) under conventional and organic cultivation systems. The above crops were established on 25 April 2019 with a plant density of 6 plants m^−2^, while the harvesting period commenced on 15 June 2019–5 August 2019. Additionally, the common bean plants were drip-irrigated using separated irrigation networks for the organic and conventional plots, while the farming practices concerning weed control and plant protection, were according to the protocols for organic farming in both cultivation systems.

The basal dressing of conventional common bean crop included inorganic fertilizer (11-15-15) which provided 55 kg ha^−1^ N. In addition to basal dressing, the common bean plants were also fertigated, from flowering to the end of the cultivation period, by applying a nutrient solution (3 mmol L^−1^ N and 5.1 mmol L^−1^ K) through a drip irrigation system, which provided a total amount of 20 kg ha^−1^ N during the whole cropping period. In the organically cultivated common beans, the basal dressing included sheep manure originating from certified organic farms according to Council Regulation (EC) No 834/2007, which provided 40 kg ha^−1^ N, and 750 kg ha^−1^ Patentkali. Unlike in the conventional bean crop, the organically cultivated plots were not fertigated. As a result, the total N inputs during summer cultivation period in organic and conventional common bean crop was 40 and 75 kg ha^−1^ N, respectively.

### 4.3. Samplings, Measurements and Methods

At the peak of harvest period, fresh common bean pods were collected when they had reached their commercial size. Immediately after sampling, randomly selected pods destined to be used for the determination of their biochemical compounds and sugar content, were stored at −80 °C, while the remaining pods were characterized for their morphological properties and then further analyzed for their nutrient profile.

#### 4.3.1. Yield, Physical Pod Quality and Nutrient Concentrations

The harvesting of common beans commenced when the fresh pods reached marketable size [63] and was repeated weekly. In total, the common bean crops were harvested eight times. At each harvesting date, the number of pods (N plant^−1^), and their total fresh weight were recorded. Subsequently, using this data, the mean fresh pod weight (g pod^−1^) was calculated. Furthermore, data from ten fresh common bean pods per treatment replication were recorded and values were averaged for the following morphological characteristics as described by Escribano et al. [64]: pod length (PL, exterior distance from the pod apex to the top) (cm), suture string (SS, straight distance from the pod apex to the top) (cm), curvature (PL/SS), width (cm), and seed number per pod. In addition, the fresh pods were oven-dried at 65 °C for 5 days and the percentage of pod dry matter (DMC) was recorded. The dried pods were ground and the obtained powder was dry ashed at 550 °C for 8 h, and the ash was dissolved in 0.5 N HCl. The above plant extract was used for determination of P (Murphy–Riley method) [65] and K (flame photometer method, Sherwood Model 410, Cambridge, UK), while in the same extract the concentrations of the macro- and micronutrients Ca, Mg, Zn, Fe, Cu and Mn were assessed using an atomic absorption spectrophotometer (AA-7000, Shimadzu Co., Tokyo, Japan). Finally, the powder material was also used for the determination of total N content in dried common bean pods by applying the Kjeldhal method [66] (Kjeltec™ 8200 Auto Distillation unit, FOSS A/S, Hillerød, Denmark).

#### 4.3.2. Biochemical Compounds Content

##### Extraction Method

Ten fresh common bean pods per treatment replication were freeze-dried and ground into fine powder. Subsequently, 50 μg of each sample were dissolved in 1 mL of pure methanol. Then the Eppendorf tubes were placed into a sonication bath at 5 °C in dark conditions for 40 min. In addition, the samples were centrifuged for 10 min at 11,000 rpm and the supernatants were collected. The above extraction method was repeated twice, and the obtained supernatants were merged and stored at −20 °C. Finally, the supernatant was evaporated using a speed vacuum and the pellets redissolved in an appropriate volume of pure methanol to concentrate the samples to 1 mg pod biomass dry weight ml^−1^.

##### Antioxidant Assays

The antioxidant properties of the above common bean extracts were determined through ferric reducing antioxidant power (FRAP) [67] and Trolox-equivalent antioxidant capacity (TEAC) [68] methods. To apply the FRAP assay, 10 μL of extract was diluted to 190 μL of FRAP reagent (chemical composition) and the absorbance was read at 593 nm. Ascorbic acid was used for the standard curve and the results were expressed in mg ascorbic acid equivalent g^−1^ dry biomass. For the TEAC assay, a freshly prepared ABTS+ reagent (7 mM ABTS, 2.45 mM potassium persulfate and 4 mM ammonium molybdate) was left at room temperature in dark condition for 16 h until a dark blue color was developed and then diluted with distilled water to give an absorbance of 0.7 ± 0.02 at 734 nm. Subsequently, 10 μL of the extract was mixed with 200 μL ABTS+ and incubated for 10 min in the dark at 30 °C. The absorbance was read at 734 nm against a methanol/water extract blank. To prepare the standard curve, Trolox was diluted in pure methanol to various concentrations and the results were expressed in mg Trolox equivalent g^−1^ dry biomass. The percentage of ABTS+ inhibition was calculated as follows:

(%) Inhibition = [(Abs control − Abs sample)/Abs control] × 100(1)

##### Total Phenolic Content (TPC) Determination

The total phenolic content of common bean pods was estimated using the Folin–Ciocalteu reagent method as described by Jan et al. [69]. In particular, 10 μL of the extract, 95 μL of 10% Folin–Ciocalteu and 95 μL of 0.5 M Na_2_CO_3_ solution were added in a microplate well. The samples were held for 120 min at room temperature in dark conditions and the absorbance was read at 765 nm. Gallic acid diluted in methanol was used as a standard solution for the curve preparation and the results were expressed in mg gallic acid equivalent g^−1^ dry biomass.

##### Total Flavonoid Content (TFC) Determination

For the determination of total flavonoid content in common bean plant extracts, the aluminum chloride colorimetric method was applied as described by Safafar et al. [70], with slight modifications. Particularly, in a microplate well, 20 μL of the pod extract were mixed with 160 μL of acidified methanol (5% *v*/*v* acetic acid) and incubated for 5 min. Then, 20 μL of 2% AlCl_3_ was added, and after incubation of the mixture for 30 min in the dark, the absorbance was recorded at 415 nm. For the standard curve, stock solution of quercetin diluted in methanol was used, while the results were expressed in mg quercetin equivalent g^−1^ dry biomass.

##### Sugars and Starch Content Determination

To investigate the impact of the different farming practices on the sugar content of common bean pods, glucose, fructose and sucrose concentrations were determined through enzymatic assays as described by Spackman and Cobb [71]. For the extraction, 20 μg of freeze-dried powdered biomass of common bean pods was dissolved in 0.75 mL ethanol 80% *v*/*v*, and the mixture was placed in a sonic bath at 25 °C for 40 min. Subsequently, the samples were centrifuged, and the supernatant was stored at −20 °C. The above extraction method was repeated twice, and the obtained supernatants were merged into one sample. The remaining pellet was dried using a speed vacuum and stored at −20 °C to determine starch content. The enzymatic determination of the above-mentioned sugars is based on reduction of NAD to NADH, of which the absorbance was measured at 340 nm.

For glucose determination, 20 μL of plant extract were added in a well of a UV microplate. Then, 200 μL of master mix were added (50 mM HEPES, 1.2 U G6PDH, 2 μmol ATP, 15 μmol Mg^2+^ and 1.2 μmol NADP) and 2 μL of hexokinase (250 U/mL) were also added. The plate was shaken automatically within the reader (Anthos Zenyth 200; Biochrom, Cambridge, UK) and the absorbance (A1) was taken at 340 nm after 60 min, until establishment of a baseline. For fructose determination, 2 μL of PGI (584 U/mL) was added in the same well, which converted fructose to glucose and concomitantly reduced the excess of NADP to NADPH. Subsequently, the absorbance was measured at 340 nm until establishment of a baseline. Finally, for sucrose determination, the above procedure was repeated by adding 2 μL of Invertase (2500 U/mL) and the absorbance (A3) was read after 90 min. For the quantification of the sugars, a standard curve was prepared including variable concentrations of glucose.

The starch content in common bean pods was determined as described by Smith and Zeeman [72]. The dried pellets were dissolved in 0.3 mL distilled water and incubated for 3 h at 120°C. Then, 0.5 mL of an enzyme mix (200 mM Na acetate, 12 U ml^−1^ Amyloglucosidase and 2 U ml^−1^ α-amylase) was added and the samples were incubated at 37 °C overnight for hydrolysis of starch to glucose. The above procedure was repeated twice, and the supernatants were merged into one sample. The glucose concentration in the obtained solutions was determined by applying an enzymatic assay where hexokinase and glucose 6-phosphate dehydrogenase were used to convert glucose to 6-phosphogluconate with concomitant reduction of NAD to NADH. Glucose was also used as a standard solution for starch quantification and starch content was expresses as mg anhydroglucose equivalent g^−1^ dry biomass by multiplying the recorded values by 162/180 (mass of anhydroglucose and glucose, respectively).

### 4.4. Statistical Analysis

In the current study, one-way ANOVA analysis was applied to identify the main effects of the three different farming practices on the quality characteristics of common bean pods. The treatment means were separated by applying the Duncan’s multiple range test when the one-way ANOVA analysis was significant at *p* ≤ 0.05. Principal component analysis (PCA) was performed on the entire data set. The ANOVA and PCA analyses were calculated using the SPSS software package.

## 5. Conclusions

The organic crop rotation of winter broccoli followed by common beans during the summer had a partially positive impact on the quality performance of common bean fresh pods. In particular, the enhanced dry matter and bioactive compound content was accompanied by reductions in pod size, mean pod weight, and number of pods per plant. Hence the total yield performance proved to be closely related to the extent of restriction in soil N availability. However, the above trend was not recorded in organic beans when the organic fertilization management involved green manure application. Specifically, the cultivation of faba beans as green manure crop during the preceding winter cultivation period boosted both the yield and the pod physical traits of the subsequent organic common bean crop to conventional levels. Furthermore, the yielded pods exhibited an improved nutrient profile compared to those derived not only from SM-treated organic beans with organic broccoli as preceding crop but also from standard conventional fertilization management.

## Figures and Tables

**Table 1:** Impact of organic farming following two different fertilization practices as compared to conventional farming on common bean pod number per plant, mean pod fresh weight (ΜFW), pod length (PL), suture string (SS), curvature (SS/PL), pod width (PW), seed number per pod (SNP) and dry matter content (DMC) of pods. CON denotes conventional farming, SΜ denotes sheep manure, GM denotes green manure and BR denotes broccoli residues.

Treatment	Pods	MFW	PL	SS	Curvature	PW	Seed	DMC (%)
	(N/Plant)	(g)	(cm)	(cm)	(SS/PL)	(cm)	(Ν/pod)	
CON	56.8 a	9.90 a	14.8 ab	18.6 a	1.25	1.37 b	6.73 a	9.86 b
O (SM + BR)	46.2 b	9.63 b	14.1 b	17.9 b	1.28	1.37 b	6.21 b	10.83 a
O (SM + GM)	53.8 a	9.94 a	15.5 a	18.9 a	1.22	1.42 a	6.88 a	9.67 b
Statistical significance	*	*	*	*	NS	*	*	***

Means from different farming practices (*n* = 8) followed by different letters within the same column indicate statistically significant differences according to Duncan multiple range test at *p* < 0.05. *, *** denote significance at *p* < 0.05 and *p* < 0.001, respectively. NS = not significant.

**Table 2:** Impact of the three different farming practices on total antioxidant activity (FRAP and TEAC) and total phenolic (TPC) and flavonoid content (TFC) in freeze-dried pods of common beans. CON denotes conventional farming, SΜ denotes sheep manure, GM denotes green manure and BR denotes broccoli residues.

Treatment	FRAP (Asc mg/g)	TEAC (Trolox mg/g)	TPC (Gallic mg/g)	TFC (Querc. mg/g)
CON	1.68 b	2.67 b	7.14 b	9.68 b
SM + BR	2.05 a	3.17 a	8.25 a	13.94 a
SM + GM	1.90 ab	3.03 ab	6.84 b	8.89 b
Statistical significance	*	**	*	**

Means for different farming practices (*n* = 8) within the same column followed by different letters indicate statistically significant differences according to Duncan multiple range test at *p* < 0.05. * and ** denote significant differences at *p* < 0.05 and 0.01, respectively.

**Table 3:** Impact of the three different fertigation schemes on sugar and starch content of dry common bean pods. CON denotes conventional farming, SΜ denotes sheep manure, GM denotes green manure and BR denotes broccoli residues.

Treatment	Sugars (mg/g)			Starch (mg/g eq. to Ahnydro Glycose)
	Glucose	Fructose	Sucrose	
CON	22.5	11.7	1.73	82.0
SM + BR	23.2	11.3	1.52	79.0
SM + GM	22.0	12.6	1.82	79.9
Statistical significance	NS	NS	NS	NS

Means of different farming practices (*n* = 8) according to Duncan multiple range test. NS = no significant.

**Table 4:** Physical and chemical properties of the soil at the experimental field.

Parameter	Value	Parameter	Value
Clay	20%	K ^2^	478 mg kg^−1^
Silt	14%	Ca ^2^	3.88 g kg^−1^
Sand	66%	Mg ^2^	1.36 g kg^−1^
pH	7.7	Fe ^1^	10.2 mg kg^−1^
Electrical conductivity	710 μS cm^−1^	Cu ^1^	2.54 mg kg^−1^
Organic matter	5%	Zn ^1^	5.10 mg kg^−1^
Total CaCO_3_	16.0%	Mn ^1^	7.84 mg kg^−1^
Total N	0.20%	B ^1^	1.06 mg kg^−1^
P ^1^	153.5 mg kg^−1^		

^1^ Available, ^2^ Exchangeable.

**Table 5:** An overview of the experimental treatments applied in both experimental years.

Winter Cultivation Period	Summer Cultivation Period	
Organic broccoli	(a) →(SM + BR)	Organic common bean
Green manure	(b) → (SM + GM)	
Conventional broccoli	(c) → (CON)	Conventional common bean

## Data Availability

Not applicable.

## Supplementary Materials

The following supporting information can be downloaded at: https://www.mdpi.com/article/10.3390/plants12010032/s1. Table S1: Eigenvalues, proportion of total variability and correlation between the 24 variables and the first two principal components (PCs); Table S2: Monthly maximum (Tmax), average maximum (MTmax), average minimum (MTmin), and minimum (Tmin) and precipitation (RR) during the experimental period (September 2018–August 2019) in Athens, Greece.
