# Supplementary material for: Fresh Pod Yield, Physical and Nutritional Quality Attributes of Common Bean as Influenced by Conventional or Organic Farming Practices
Source: Plants (Basel). 2022 Dec 21;12(1):32. doi: 10.3390/plants12010032 (PMC9824475; doi:10.3390/plants12010032)
Supplement: Supplementary file 1 [file plants-12-00032-s001.zip › Supplementary material.pdf]

Table S1. Eigenvalues, proportion of total variability and correlation between the 24 variables and the first two principal components (PCs).

**Variable**	**PC1**	**PC2**
Pod N	0,756	-0,655
FW	0,954	-0,298
PL	1,000	0,016
SS	0,991	-0,137
Curvature	-0,984	-0,178
Width	0,831	0,556
Seeds	0,977	-0,213
DMC	-0,960	0,279
N	0,907	-0,422
P	-0,125	0,992
K	0,438	0,899
Ca	0,921	0,389
Mg	0,669	0,743
Fe	0,661	0,751
Mn	0,757	0,653
Cu	0,756	0,654
Zn	0,037	0,999
FRAP	-0,493	0,870
TEAC	-0,340	0,940
TPC	-0,973	0,232
TFC	-0,958	0,287
GLU	-0,999	0,050
FRU	0,961	0,275
SUC	0,990	-0,140
Starch	0,386	-0,922
Eigenvalue	16,182	8,818
Variance %	64,728	35,272
Cumulative %	64,728	100

Table S2. Monthly maximum (Tmax), average maximum (MTmax), average minimum (MTmin), and minimum (Tmin) and precipitation (RR) during the experimental period (September 2018–August 2019) in Athens, Greece.

Year	Month	Mtmax (°C)	Tmax (°C)	Mtmin (°C)	Tmin (°C)	RR (mm)
2018	Sep	29.2	36.3	21	16.1	43.8
	Oct	23.3	26.8	16.3	9.4	0.6
	Nov	19.2	28.1	12.9	7.6	51.6
	Dec	14.2	19.3	7.9	3.7	73.7
2019	Jan	12.8	16.8	6.5	-0.1	125
	Feb	13.5	19.1	6.9	3.3	59.2
	Mar	17.6	24.1	9.6	5.3	26.8
	Apr	19.1	25.8	11.8	8	115
	May	24.2	30.2	15.8	11.2	2.2
	Jun	31	35.2	22.7	17.9	2.6
	Jul	32.6	36.4	23.8	20.9	1
	Aug	33.6	36.3	25.6	22.6	0
